# Expression Profile of Human Cytomegalovirus *UL111A* cmvIL-10 and LAcmvIL-10 Transcripts in Primary Cells and Cells from Renal Transplant Recipients

**DOI:** 10.3390/v17040501

**Published:** 2025-03-31

**Authors:** Giovana W. C. Almeida, Martha T. Oliveira, Isabella G. L. Martines, Giuliano C. Fiori, Michael M. Nevels, Ian J. Groves, John Sinclair, José Medina-Pestana, Rayra Sampaio da Silva, Monica Nakamura, Lucio Requião-Moura, Emma Poole, Maria C. Carlan da Silva

**Affiliations:** 1 Centro de Ciências Naturais e Humanas (CCNH), Universidade Federal do ABC (UFABC), São Bernardo do Campo 09606-070, SP, Brazil; gih_carneiro@hotmail.com (G.W.C.A.); marthatoliveira@gmail.com (M.T.O.); isabellaglmartines@gmail.com (I.G.L.M.); giuliano.decastro@gmail.com (G.C.F.); 2 School of Biology, University of St. Andrews, St. Andrews KY16 9ST, UK; mmn3@st-andrews.ac.uk; 3 Molecular Medicine, Cleveland Clinic, Cleveland, OH 44106, USA; grovesi@ccf.org; 4 Department of Medicine, University of Cambridge, Cambridge CB2 1TN, UK; js152@cam.ac.uk; 5 Hospital do Rim, Fundação Oswaldo Ramos, Vila Clementino 04038-002, SP, Brazil; medina@hrim.com.br (J.M.-P.); rayra.silva@hrim.com.br (R.S.d.S.); monica.nakamura@hrim.com.br (M.N.); lucio.requiao@gmail.com (L.R.-M.); 6 Nephrology Division, Universidade Federal de São Paulo, Vila Clementino 04021-001, SP, Brazil; 7 Department of Pathology, University of Cambridge, Cambridge CB2 0QQ, UK

**Keywords:** human cytomegalovirus (HCMV), *UL111A* transcripts, HCMV IL-10, HCMV latent infection, HCMV lytic infection, kidney transplant

## Abstract

Human cytomegalovirus (HCMV) is a high-risk pathogen in immunocompromised individuals, especially in transplant recipients. HCMV viremia must be monitored, and frequently, patients are treated with antiviral agents. HCMV has a variety of strategies to modulate host antiviral responses, and one important player is a viral homolog of the cellular interleukin-10 (cIL-10). The viral *UL111A* gene produces several HCMV IL-10 transcripts and protein isoforms through alternative splicing. The cmvIL-10 (isoform A) has similar properties to cIL-10, while LAcmvIL-10 (isoform B) has more restricted biological properties. Other isoforms are produced (C to H) but have unknown functions. Here, we investigated the expression of the most abundant transcripts, cmvIL-10 and LAcmvIL-10, in productively and latently infected cells and in peripheral blood mononuclear cells from renal transplant recipients up to 60 days post-transplantation. This study investigated HCMV cmvIL-10 and LAcmvIL-10 transcription profiles in vitro, in productive and latent infection, and in vivo, in peripheral blood mononuclear cells (PBMCs) of renal transplant patients. In vitro, both cmvIL-10 and LAcmvIL-10 transcripts were detected in both types at high levels and low levels in MRC-5 and latent infected (CD14+). When PBMCs from transplant patients were analyzed, LAcmvIL-10 was detected mostly sporadically and in only a few patients, while cmvIL-10 was found in all patients at all time points. Furthermore, it was observed in PBMCs that expression of cmvIL-10 was positively associated with an increase in viral DNA detection in the subsequently collected sample, indicating that expression of cmvIL-10 might precede viral DNA replication. These results contribute to the understanding of HCMV biology in different phases of infection. In addition, our initial analysis suggests that monitoring cmvIL-10, along with viral DNA, could improve early detection of HCMV reactivation in transplant recipients.

## 1. Introduction

Human cytomegalovirus (HCMV) is a highly prevalent betaherpesvirus [1]. Primary infections are commonly asymptomatic due to the immune system’s robust control of viral replication. Afterward, the virus remains in a latent state and can sporadically reactivate. Reactivation does not usually cause disease in healthy individuals, but in immunocompromised hosts, the virus can lead to severe disease and death [2]. HCMV is especially a threat to transplant recipients. Symptomatic HCMV reactivation is associated with organ rejection and secondary infections. Recipients who are HCMV seronegative have a higher risk of serious complications caused by primary HCMV infection, including graft rejection and mortality [3]. In kidney transplant recipients, HCMV reactivation or primary infection transmitted by the graft can lead to clinical complications, such as increased risk of rejection, chronic nephropathy, and death. Therefore, HCMV viremia is constantly monitored post-transplantation, and high-risk transplant recipients usually receive antiviral prophylaxis [4].

HCMV can infect many cell types in the host, but permissiveness to viral replication can vary according to the cell type. During a productive infection in permissive cells, all classes of lytic viral genes (immediate-early, early, and late) are expressed, and viral particles are produced and released. In contrast, during viral latency, in non-permissive cells, the viral genome persists in the absence of lytic replication and virus production. The virus can reactivate under specific conditions, including cellular differentiation and inflammation-associated signaling [5,6,7]. In fact, inflammatory cytokines induce viral reactivation, and this process is important during organ transplantation, where substantial inflammation occurs [8].

The most characterized sites of HCMV latency in vivo are bone marrow resident CD34+ myeloid progenitor cells and their derivative CD14+ monocytes present in peripheral blood [9,10]. Reactivation in these cell types is characterized by reinitiation of viral replication following the differentiation of precursor cells into macrophages or dendritic cells [11,12]. In experimental models of latency, viral transcripts represent a minority of total transcripts, especially transcripts encoding lytic cycle proteins. Among the genes associated with latency, which probably help maintain the latent genome and are detected at higher levels in vitro, as well as having been detected in natural latency, are *UL138*, *US28*, *UL144*, *UL81-82ast* (LUNA), and *UL111A* (viral IL-10) [13,14,15].

The immune system is critical to controlling infection, and HCMV is one of the first viral agents to be detected in immunosuppressed transplant patients, highlighting the fact that viral replication and production of viral particles are restricted by the immune system [16]. However, despite the control by the immune system, HCMV has numerous strategies to avoid viral clearance in any mode of infection, from lytic to persistent or latent. One important protein in this process is the viral homolog of human interleukin-10 (viral IL-10) encoded by the *UL111A* gene [17]. The *UL111A* gene is known to be differentially spliced and produces cmvIL-10 (isoform A) and LAcmvIL-10 (isoform B) proteins of 175 and 139 amino acids, respectively. Expression of cmvIL-10 was demonstrated during lytic infection in fibroblasts. The protein acts through the cIL-10 receptor and has immunomodulatory properties similar to cIL-10 [18,19,20]. LAcmvIL-10 was initially identified in latently infected cells and later during lytic infection [21,22], and it has restricted immunomodulatory properties [23]. Six other transcripts, resulting from alternative splicing of the *UL111A* region, have been reported to be expressed in productively infected MRC-5 cells. These transcripts were designated C, D, E, F, G, and H and have unknown functions [24,25].

To date, studies investigating the expression pattern, quantity, and kinetics of HCMV IL-10 transcripts during latency and reactivation, both in vitro and in vivo, are scarce. Such data could provide information on how the virus manipulates the immune system in different states of infection. In addition, the data could be informative for monitoring the progression of HCMV infection and identifying patients at risk of HCMV disease since viral IL-10 has a severe potential to impair the host immune response.

In this work, we show by RT-qPCR the expression of the most abundant and characterized HCMV IL-10 transcripts, cmvIL-10 and LAcmvIL-10, in productively infected primary MRC-5 cells and during the establishment of latency in CD14^+^ monocytes in vitro. Most importantly, the expression pattern and quantitative levels of these transcripts were determined in vivo in peripheral blood mononuclear cells (PBMCs) isolated from kidney transplant recipients periodically up to 60 days post-transplantation. The expression levels of both transcripts were compared with intracellular viral DNA levels in PBMCs and viral load in the plasma of the patients. Furthermore, we investigated the existence of a relationship between the expression of HCMV IL-10 transcripts and intracellular viral DNA load. As the analysis suggests, the transcription profiles differ in vitro and in vivo. During permissive infection in vitro, both transcripts were detected, with LAcmvIL-10 expression even surpassing cmvIL-10 levels. In vivo, however, while LAcmvIL-10 was mostly detected sporadically, cmvIL-10 expression was found in all patients throughout the experiment. In addition, our initial data suggest that elevated cmvIL-10 expression may contribute to or predict higher viral replication over time in vivo. Therefore, investigation of cmvIL-10 expression, together with viral DNA, could help in monitoring viral reactivation and possible development of disease in transplant patients.

## 2. Materials and Methods

### 2.1. Cells and Viruses

The MRC-5 human embryonic lung fibroblast cells (ATCC-CCL-171) were maintained in Dulbecco’s Modified Eagle’s Medium (DMEM) with 10% fetal bovine serum (FBS), 100 U/mL penicillin, and 100 μg/mL streptomycin. Primary CD14+ monocytes were isolated from peripheral blood (NHSBT, Cambridge or NHSBT, Colindale, London, UK), as previously described [12]. Cells were incubated at 37 °C in a humid atmosphere with 5% CO_2_.

Two viruses, derived from the clinical TB40E HCMV strain, cloned as Bacterial Artificial Chromosomes (BACs), were used: the TB40E strain [26] and the TB40E-IE2YFP [27,28]. For viral production, the BAC DNAs were electroporated into MRC-5 cells, and after a complete cytopathic effect, supernatants were collected. Infectious yields were determined by plaque assay on MRC-5 cells.

### 2.2. Cell Infections

MRC-5 cells were infected with the HCMV TB40E strain at a multiplicity of infection 1 (MOI 1) for 2 h with subsequent media replacement. At the indicated time points, cells were collected for RNA extraction. CD14+ were infected with strain TB40/E-IE2-EYFP at MOI 5 for 3 h, followed by media replacement and further incubation until 120 h to allow latency to establish. For viral reactivation, phorbol myristate acetate (PMA) (50 ng/μL) was added to the media at 96 hpi and left for 24 h. At 120 hpi, RNA was harvested.

### 2.3. Samples from Kidney Transplant Recipients

The kidney transplant recipients’ blood samples were collected from patients admitted to the Hospital do Rim-São Paulo (Fundação Oswaldo Ramos) in 2022–2023. All donors were HCMV seropositive and transplant recipients consisted of 15 seropositive (D+/R+) and 2 seronegative (D+/R−). All patients received a routine regimen of immunosuppressive drugs, at the day of surgery, consisting of tacrolimus or cyclosporine with mycophenolate mofetil and steroids. The dosage of immunosuppression and the treatment of rejection episodes were established according to specific protocols. All individuals undergoing transplantation signed the informed consent form (ICF), which will be stored at the hospital for 10 years.

Peripheral blood samples were collected at different times: the first collection was carried out on the day of transplantation and immunosuppression, prior to these procedures (1st day), and the subsequent collections were on the 7th, 21st, 45th, and 60th day after transplantation. Blood samples were also collected when the virus was detected for the first time (cmv+), at the beginning (Initial Treatment-IT), and at the end of ganciclovir treatment (Final Treatment-FT).

### 2.4. Serology

Serum anti-HCMV IgG was tested using the commercial Biolisa CMV Citomegalovirus Igg kit–K112-1 (Bioclin) (Belo Horizonte, MG, Brazil), according to the manufacturer’s instructions. The limit of detection was >15 UA/mL.

### 2.5. Monitoring of Viral Load in Plasma Samples

Quantitation of viral DNA in the plasma was performed using the Abbott RealTime CMV assay by Hospital do Rim-São Paulo. HCMV replication was defined by DNAemia with a threshold > 34.5 IU/mL. Analyses began to be carried out from the 21st day after transplantation or according to the patient’s symptoms. Patients with a viral load above 5000 IU/mL underwent antiviral treatment with ganciclovir.

### 2.6. Clinical Evaluation and Antiviral Therapy

The strategy used for reducing the risk of CMV was the pre-emptive strategy [29]. Antiviral treatment was initiated in asymptomatic patients when viral load exceeded 5000 IU/mL or with any viral load in symptomatic patients or seronegative asymptomatic patients. HCMV infection was defined with the detection of HCMV DNA in blood (viremia) and considered asymptomatic when it occurred without clinical symptoms and symptomatic when abnormal laboratory findings occurred concomitantly with clinical symptoms, which included fever, asthenia, myalgia, leukopenia, thrombocytopenia, or alterations in liver enzymes, or invasive disease, in which there was evidence of viral inclusion in organ or tissue cells [30]. Treatment was performed with intravenous ganciclovir, and the duration of treatment varied with the evolution of the viral load, being discontinued when the patient achieved a viral load < 200 IU/mL.

### 2.7. Peripheral Blood Mononuclear Cells (PBMCs) Isolation

Blood samples (5 mL) were diluted 1:1 in Hank’s Balanced salt solution (HBSS) 1X without calcium and magnesium (Gibco) (Grand Island, NY, USA), underlaid with Ficoll-Paque and centrifuged. PBMCs were collected from the interface, washed 2X with HBSS 1X, and the cell pellet was resuspended in 200 µL of (HBSS) 1X.

### 2.8. Acid Nucleic Extraction and cDNA Synthesis

DNA/RNA was extracted using Virus RNA+DNA Preparation Kit (Cellco; Cat. DPK-115S) (São Carlos, SP, Brazil), treated with the DNase I–Amplification Grade (Sigma-Aldrich) (Saint Louis, MO, USA), and converted to cDNA using the SuperScript™ II Reverse Transcriptase (ThermoFisher Scientific) (Carlsbad, CA, USA), as per manufacturer’s protocols using 8 µL of RNA as a template per reaction.

### 2.9. Primer Design and RT-qPCR Optimization

Primers for the viral transcripts *UL123* and *UL138* and for the cellular glyceraldehyde-3-phosphate dehydrogenase (GAPDH) used in CD14+ have been described [31]. Amplification of the HCMV IL-10 transcripts in in vitro assays (MRC-5 and CD14+ cells) was performed with a SYBR Green relative expression for GAPDH RT-qPCR assay. Amplification of viral *UL44* DNA in PBMCs isolated from transplant patients was used to determine intracellular viral DNA levels and was also performed with a SYBR Green relative expression for GAPDH RT-qPCR assay; amplification of the HCMV IL-10 transcripts in PBMCs however, was performed with a TaqMan absolute expression RT-qPCR assay. Primers and probes were designed based on the Boundary spanning method (BSP) [32] and are depicted in Table S1 and Figures S1 and S3.

HCMV IL-10 transcripts primer specificity in a SYBR Green assay was tested using plasmids (pEF-1) containing ORFs to the corresponding transcripts donated by Dr. Mengtao (University of California, Los Angeles) [24]. Plasmid 10-fold dilutions were made from an initial sample of known concentration, and dilutions 10^−5^ and 10^−4^ were used in qPCR. The specificity of the primers was confirmed by Melting Curve analysis. Specific amplification of transcripts cmvIL-10 and LAcmvIL-10 generated Tm values of 86.19 °C and 86.46 °C, respectively (Figure S2).

To verify primer specificity in TaqMan assays, reactions were made using the plasmids pEF-1, containing the HCMV IL-10 ORFs, at different concentrations (10^1^ to 10^5^ copies per µL). Figure S4 shows the Ct values obtained after amplification of cmvIL-10 and LAcmvIL-10, demonstrating that the assays are specific for each transcript.

### 2.10. Standard Curve for Absolute Quantification UL111A Transcripts in PBMC

For the standard curve, plasmids containing the ORFs for cmvIL-10 and LAcmvIL-10 were used. The concentration of plasmid DNA was measured in ng/µL using the Biodrop Duo (Biochrom™). To determine the number of copies of interest (molecules/µL), the following formula was used:

number of copies of interest = DNA concentration × 6.02 × 10²³*÷(number of bp × 660)

where DNA concentration (g/µL) is multiplied by Avogadro’s number and divided by the total molecular weight, which is calculated as the number of base pairs multiplied by the average molecular weight of a base pair (bp) (660 g/mol). Based on the results, an initial dilution was performed to reach 10^5^ molecules/µL, and 10-fold serial dilutions were performed until reaching 10^0^.

### 2.11. Quantitative Reverse Transcription PCR (RT-qPCR) Analysis

RT-qPCR reactions, using SYBR Green, consisted of 5 µL of Master Mix (GoTaq RT-qPCR KIT, Promega) (Madison, WI, USA), 1 µL of primer Forward (F) (2.5 mM), 1 µL of primer Reverse (2.5 mM), 0.1 µL of CRX, 1.9 µL of H_2_O, and 1 µL of sample. Amplification conditions consisted of 95 °C for 2 min, followed by 45 cycles of 95 °C for 3 s and temperature defined by the primers for 30 s. For *UL44* primers, the annealing temperature was 60 °C; for cmvIL-10 primers, the temperature was 66 °C; and for LAcmvIL-10 primers, the temperature was 70 °C.

For TaqMan assays, the reactions consisted of 5 µL of Master Mix (TaqMan Fast Advanced Master Mix, Applied Biosystems) (V. A. Graiciuno, Vilnius, Lithuania), 1.5 µL of primer Forward (F) (2.5 mM), 1.5 µL of primer Revere (R) (2.5 mM), 0.5 µL of probe (5µM), 0.5 µL of H_2_O, and 1 µL of sample. The following amplification conditions were used: 50 °C for 2 min, 95 °C for 20 s, followed by 45 cycles of 95 °C for 2 s and 60 °C for 20 s. All reactions were performed in triplicate.

Relative expression in SYBR Green assays was calculated by the Delta-Delta Ct (ΔΔCT) method. First, ΔCt for the HCMV target is calculated by normalizing it against GAPDH, following the formula:

ΔCt = Ct _target HCMV_ − Ct _GAPDH_

Next, the HCMV target was normalized to the control by calculating ΔΔCt:

ΔΔCt = ΔCt _HCMV target_ − ΔCt _control_

Finally, to determine the fold change in gene expression of the target relative to the control, the following formula was used:

Relative expression = 2^−ΔΔCt^

The abundance of each product in TaqMan was determined by comparison with a standard curve generated from qPCR analysis of 10-fold serial dilutions, in the range of 1 to 10^5^ copies, of plasmids (pEF-1) containing the sequences for cmvIL-10 and LAcmvIL-10. In all analyses, 10^2^ or 10^3^ copies of plasmids encoding the unspliced *UL111A* gene were used as controls for nonspecific amplification. The detection limits of the transcripts were similar, being approximately 10 copies/µL.

### 2.12. Statistical Analysis

Transcript copy numbers were transformed by Log_10_(X+1), and ANCOVA (analysis of covariance) was performed analyzing cmvIL-10 and viral DNA (quantification data from before ganciclovir treatment), using both data from the same sample (same time point) and cmvIL-10 quantification data and viral DNA data from the subsequent collection point (sequential collection). Pr(>F) <  0.05 was considered statistically significant. Statistical analyses and graphs were made using both R statistics and GraphPad Prism version 5.0 software.

## 3. Results

LAcmvIL-10 transcript is produced both in lytic and latent infected cells [21,22]. While cmvIL-10 has been shown to be expressed during the HCMV lytic cycle [17,18], it has also been demonstrated to play a role in the regulation of cIL-10 [15], likely preventing apoptosis in latent infected cells [33,34]. Here, we investigated the production of cmvIL-10 and LAcmvIL-10 during productive and latent infection in infected cells and during natural infection in renal transplant recipients.

### 3.1. Relative Levels of UL111A Transcripts cmvIL-10 and LAcmvIL-10 in Productive and Latently Infected Cells

**Figure 1:**
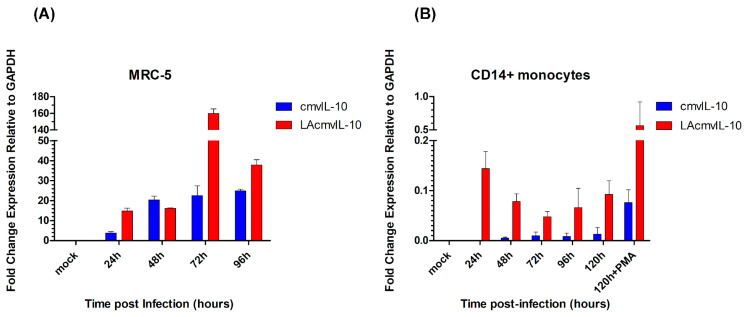
Analysis of *UL111A* cmvIL-10 and LAcmvIL-10 transcripts in permissive infected MRC-5 cells and during the establishment of latency in CD14+ monocytes. MRC-5 (**A**) and CD14+ monocytes (**B**) were infected with HCMV TB40E strain at MOI 1 and 5, respectively; cmvIL10 and LAcmvIL-10 RNA levels were quantified by RT-qPCR and are expressed as fold change relative to housekeeping gene GAPDH (on the *Y*-axis) at different time points: 24, 48, 72, and 96 hpi (on the *X*-axis). For viral reactivation in CD14+ monocytes, phorbolmyristate acetate (PMA) was added to the media at 96 hpi and left until 120 hpi. Each bar represents the means with standard deviations of fold changes in expression of the experimental results of three biological and two technical repeats for each time point (expression in CD14+ monocytes was investigated from 2 different donors, and bars represent the mean of both sets of data).

**Figure 2:**
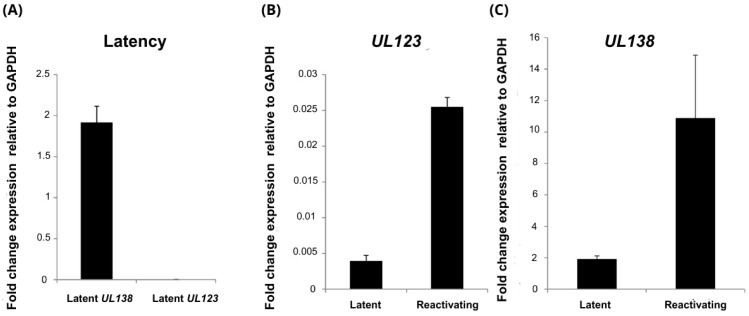
Relative expression of *UL123* and *UL138* genes in CD14+ cells. *UL123* (immediate-early gene expressed only during lytic infection) and *UL138* HCMV transcripts were quantified by RT-qPCR at 96 hpi during latency (**A**) and in the absence (latent) and in the presence of PMA treatment (reactivating) at 96 hpi (**B**) and at 120 hpi (**C**). Expression is shown as fold change relative to the housekeeping gene GAPDH of experimental results of three biological and two technical repeats (on the *Y*-axis).

In productively infected MRC-5 cells, both cmv-IL10 and LAcmvIL-10 were expressed at all time points; however, while cmv-IL10 tended to stabilize after 48 hpi, LAcmvIL-10 decreased after 72 hpi (Figure 1A). In CD14+ cells, during latency, cmvIL-10 and LAcmvIL-10 were detected at much lower levels than in lytic infection in MRC-5 cells. Viral reactivation, by differentiating cells with PMA, leads to an increase in the expression of both transcripts (Figure 1B) in CD14+ monocytes. Analyses of *UL138* (expressed during both latent and lytic infection) and *UL123* (expressed only during lytic infection, also known as IE1) transcripts in latent infected CD14+ show that while cells were expressing low levels of *UL138*, *UL123* expression was significantly repressed (Figure 2A), characterizing latency. Viral reactivation with PMA in CD14+ cells leads to increased expression of *UL123* and *UL138* (Figure 2B and Figure 2C, respectively), as expected. These results show that, in vitro, both cmvIL-10 and LAcmvIL-10 transcripts are expressed in permissive and latent infected cells at, respectively, high and low levels.

### 3.2. Patient History and Clinical Data of Transplant Recipients

Overall data relevant to patients involved in the study are shown in Table S2. Of a total of 17 patients, 15 were HCMV seropositive, and two were HCMV seronegative. Despite the seronegative status, patient 16 presented an active HCMV infection, as determined by viral DNA replication in the plasma, above threshold > 34.5 IU/mL. No DNA was detected in seronegative patient 17 during the time course of analysis. Eleven patients underwent treatment with ganciclovir, and one patient died due to organ rejection.

### 3.3. Monitoring Intracellular and Extracellular Viral DNA in Patient Samples

**Figure 3:**
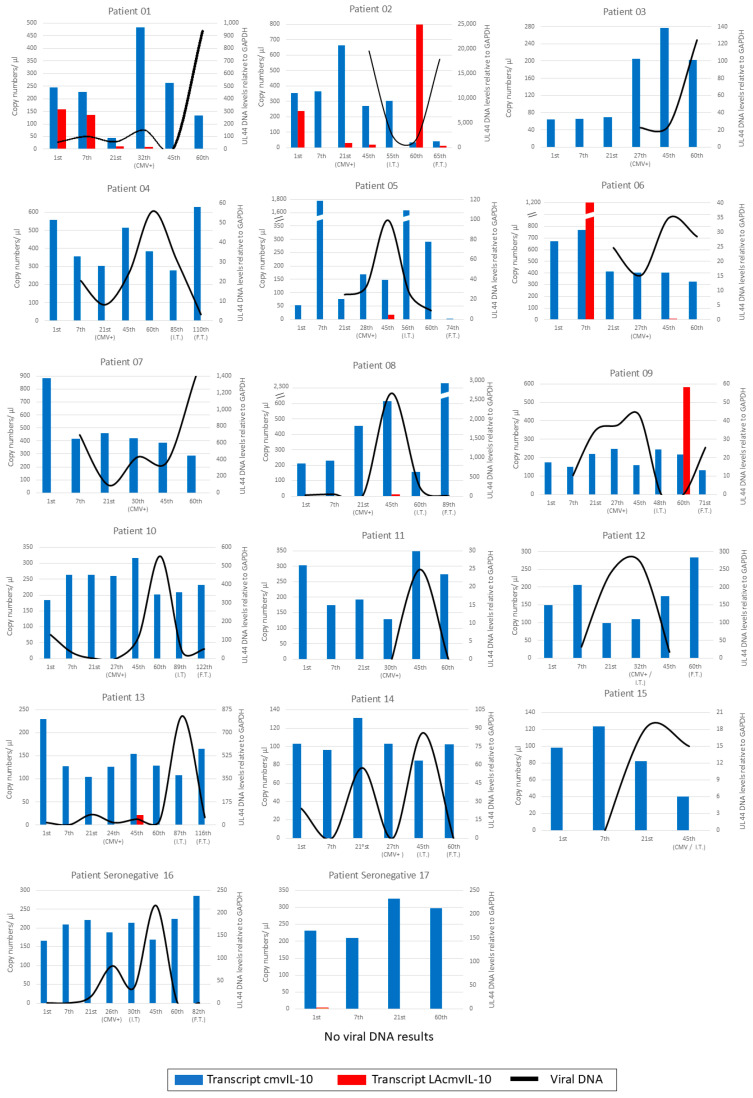
Expression of cmvIL-10 and LAcmvIL-10 transcripts and intracellular viral DNA in PBMCs from transplant recipients. In each graph, the left Y- axis shows the expression of transcripts in copy numbers/µL; the right Y-axis shows the relative levels of intracellular DNA (UL44 gene) relative to GAPDH; and the X-axis shows the time points (days) of the analysis. The transcript expressions are shown in bars: transcript cmvIL10 (blue) and LAcmvIL-10 (red). Viral DNA is shown in black lines. The beginning and end of ganciclovir treatment are indicated as I.T. (initial treatment) and F.T. (final treatment).

Results of the routine diagnosis of HCMV DNA in the plasma of patients indicate an average of detection between 20 and 30 days post-transplantation (see Abbot assay results in Figure S5). Intracellular viral DNA detection in PBMCs occurred at more variable times, and notably, in five patients (Figure S5, patients 01, 08, 10, 13, and 14), DNA was already detected at the first sample collection time point (1st day), before the administration of immunosuppressive drug mycophenolate. Generally, amplification occurred in a similar temporal pattern with plasma DNA levels, as expected. The peak of viral replication occurred in most patients around either the 45th day (*n* = 8/17) or the 60th day (*n* = 5/17). In most patients who received the antiviral ganciclovir (Table S2), treatment occurred between the 45th and 60th day, and viral DNA replication began to decrease from the 60th day onwards (*n* = 9/11) (Figure 3, patient 16). These results show a similar pattern of intracellular and plasma viral DNA detection in patient samples. Moreover, in our analysis, intracellular viral DNA was detectable in PBMCs from some patients (*n* = 6/17) before immunosuppression.

### 3.4. Kinetics of UL111A Transcripts in PBMCs of Transplant Recipients

The expression of the HCMV *UL111A* cmvIL-10 and LAcmvIL-10 transcripts in PBMCs from transplant recipients was verified by RT-qPCR assay; cmvIL-10 transcripts were detected in all samples, since the 1st day of analysis, regardless of the presence of detectable intracellular viral DNA (Figure 3). In most patients, the number of copies ranged from 100 to 250 per µL; cmvIL-10 was also detected in the seronegative patients (16 and 17), while patient 16 had viral DNA detected, and no viral DNA was detected in patient 17 (Figure 3).

As for LAcmvIL-10, expression was detected sporadically in eight patients (01, 02, 05, 06, 08, 09, 13, and 17). LAcmvIL-10 transcript was identified in seven patients (01, 02, 05, 06, 08, 09, and 13). Analyzing these patients, it was possible to observe that the B transcript appears to be more frequently expressed on the 45th day, being detected in four out of the seven patients (02, 05, 08, and 13). At the beginning of treatment with ganciclovir, this transcript was not detected in any of the patients. Despite the small sample number, which is insufficient to perform a powerful statistical analysis, LAcmvIL-10 expression appears to be inversely related to viral DNA detection, and future investigations are needed to confirm this observation.This could explain the absence of this transcript expression at the start of ganciclovir treatment, as the viral load was at its peak at that time. Notably, patient 02 exhibited LAcmvIL-10 transcript expression in a greater number of samples—five out of seven samples (1st day, 21st day, 45th day, 60th day, and final treatment day). Interestingly, viral DNA in patient 02 was only detected on the 45th day of collection.

We conclude from these analyses that cmvIL-10 is the most expressed HCMV IL-10 transcript during early reactivation to productive infection, while LAcmvIL-10 seems to generally be expressed at much lower levels than cmvIL-10.

### 3.5. Covariance Analysis

**Figure 4:**
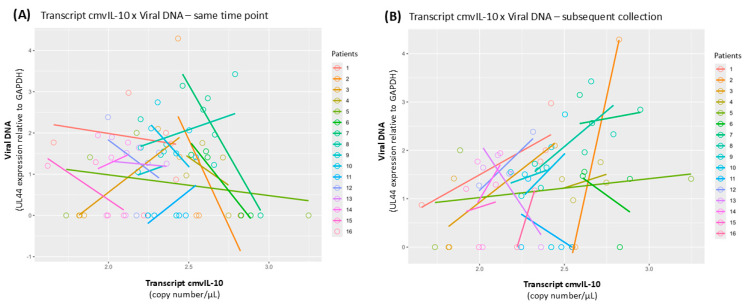
Analysis of covariance between cmvIL-10 transcript expression and viral DNA. Covariance indicates the extent to which two (random) variables are dependent on each other. A positive covariance on a graph is represented as a line that slopes upwards. (**A**) Analysis of cmvIL-10 and viral DNA of samples from the same time point, i.e., analysis was made using data from the same sample; same time-point analysis did not show a significant covariance (Pr(>F) = 0.1976). (**B**) Analysis of cmvIL-10 and viral DNA in sequential samples, i.e., analysis of cmvIL-10 transcript expression was performed with one sample, and viral DNA expression was performed with a sample from a subsequent collection time point in that patient; subsequent collection analysis showed a statistically significant positive covariance (Pr(>F) = 0.0212). Circles represent the expression of cmvIL-10 transcript (in copies/μL, on the *X*-axis) and viral DNA levels (*UL44* DNA levels relative to GAPDH, on the *Y*-axis) in the samples; lines represent the best straight line showing the relationship of cmvIL-10 concentration with viral DNA expression for each patient (each color represents a different patient). All samples were collected before ganciclovir treatment.

In the previous analysis, we generally observed a relationship between an increase of transcript cmvIL-10 and viral DNA in the time course of infection. To further verify this observation, a covariance analysis (ANCOVA) was performed between the expression of cmvIL-10 and viral DNA levels in samples collected at the same time point and cmvIL-10 and viral DNA in samples from the subsequent collection points. The analysis revealed no significant relationship between viral DNA and cmvIL-10 transcription in samples collected at the same time point (Figure 4A). However, analysis of covariance revealed that increases in the expression of cmvIL-10 were significantly associated with an increase in viral DNA levels in the subsequent sample (Pr(>F) = 0.0212) (Figure 4B). These results suggest that expression of cmvIL-10 precedes viral DNA replication, and it can be an indicator of reactivation.

## 4. Discussion

HCMV IL-10 viral proteins can potentially impact the immunosuppression of the host in different phases of infection [35]. While cmvIL-10 has immunosuppressive functions analogous to cIL-10, LAcmvIL-10 does not share all the properties of cmvIL-10 [36]. Several reports have demonstrated the expression of the HCMV *UL111A* in infected cells as well as in infected individuals; however, in these studies, there was no distinction made between the specific transcripts or protein isoforms [36,37,38,39,40,41]. Here, we evaluated the expression of the most abundant *UL111A* transcripts, cmvIL-10 and LAcmvIL-10, in infected cells in culture, in addition to PBMCs from HCMV seropositive and seronegative individuals undergoing kidney transplantation. Between those two transcripts, cmvIL-10 was the transcript with the highest levels of expression in PBMCs from transplant recipients and was detected in all patients in all time points analyzed after transplantation. Importantly, cmvIL-10 was even detected on the day of transplantation and in samples where viral DNA was not detected or was present at low levels (Figure 3).

Analysis of viral DNA demonstrated that intracellular viral DNA replication in PBMCs preceded and was associated with viral DNA in the plasma (viremia) in most cases. We did not observe a relationship between HCMV viremia and viral disease; however, this was not the focus of our work, and specific analysis as a larger number of transplant recipients would be required to generate data with statistical significance for these studies. During our study, one patient presented organ rejection, but it was not HCMV-associated (Table S2).

On average, viral DNA was detected in PBMCs approximately 21 days after transplantation, but in five patients, intracellular viral DNA was detected on the first day of analysis, before immunosuppression induced by Mycophenolate, with no evidence of production of extracellular virus in the plasma (Figure S5). It is possible that in these patients, the detection of viral DNA in PBMCs is due to viral latency in the absence of virus production in the myeloid lineage, mainly CD14+ monocytes [6,42,43]. However, it is also possible that they have low levels of viral reactivation in monocyte-derived macrophages and dendritic cells, present in the PBMC pool, due to intrinsic immunosuppression, which is controlled by the immune system. After immunosuppression, plasma viral DNA, likely originating from lysed cells from active sites of infection, started to be detected. We considered the last hypothesis more likely because viral DNA is present at very low copy numbers in PBMCs from HCMV seropositive healthy individuals, and its detection requires sensitive techniques [44]. In fact, even during events of viral reactivation in immunosuppressed transplant recipients, the cell types present in PBMCs can appear to have very low or even undetectable levels of viral DNA [45]. However, in our analysis, we detected viral DNA in PBMCs from all seropositive and one seronegative patient (this may be explained by the serology typing used). In most cases, viral plasma DNA was detected after intracellular DNA, and their levels increased concomitantly, as would be expected. Therefore, we concluded that our assay is sensitive enough to detect low levels of viral DNA replicating in PBMCs, which might be higher than plasma levels before a full reactivation episode.

cmvIL-10 transcription was detected in our work in some samples in the absence of viral DNA (Figure 3). Previous studies showed cmvIL-10 transcription during lytic infection in vitro [17,18]. Additionally, cmvIL-10 protein has been detected in the plasma of healthy HCMV seropositive individuals with no signs of disease [40,41] and in renal transplant recipients [41]. Interestingly, the HCMV IL-10 protein has also been detected in HCMV seronegative individuals [40,42]. However, as before, it was not possible to distinguish between the viral isoforms in these studies due to the absence of available specific antibodies. Importantly, we also detected cmvIL-10 transcription in an HCMV seronegative patient with viral DNA reactivation (Figure 3, Patient 16) and in an HCMV seronegative patient with no detectable viral DNA during the 60-day time course analysis (Figure 3, Patient 17). Together, these findings indicate that cmvIL-10 mRNA and protein can be expressed at significantly high enough levels to be detected when viral DNA is latent or replicating at low levels. Therefore, evaluation of cmvIL-10 expression could be considered for diagnostic tests for HCMV infection.

Although our study did not discriminate if cmvIL-10, detected in PBMCs, is expressed in latent or lytically infected cells during the early stages of reactivation, we showed here that both cmvIL-10 and LAcmvIL-10 transcripts could be detected at low levels. We also showed that those levels increase during viral reactivation in an HCMV latent infection model in CD14+ cells (Figure 1B). Therefore, most likely, cmvIL-10 is produced and has a role in latent viral infection. Indeed, in vitro studies showed that cmvIL-10 is able to inhibit apoptosis and restrict the ability of latently infected myeloid progenitors to differentiate into dendritic cells [13,35]. Regardless, our analysis demonstrated the production of cmvIL-10 mRNA from early reactivation to productive infection in infected individuals.

Although the sample size in our study was limited, our work also provides the first evidence that levels of cmvIL-10 expression in most samples tend to increase and precede high levels of viral DNA replication in the time course of analysis (Figure 4B). These initial results, associating cmvIL-10 expression with intracellular viral replication, suggest that investigating the expression of cmvIL-10 can help monitor HCMV viral reactivation. More studies investigating this matter would be beneficial in order to reiterate our data. Notably, in a recent study using samples from patients with Aspergillus infection, no correlation was found between HCMV IL-10 protein levels and HCMV viremia [39]. Different from our work, this study verified protein production while we detected mRNA levels, which is likely much more sensitive. In addition, we analyzed samples from transplant recipients who are highly immunosuppressed at different time points after immunosuppression. Nevertheless, more studies are needed to confirm our findings.

Finally, contrary to cmvIL-10, in our analysis, the LAcmv-IL10 transcript was detected sporadically and only in a few patients, contrasting to its expression in vitro in infected productive MRC-5 cells, where it is expressed at high levels and at all time points evaluated (Figure 1A). LAcmvIL-10 was first identified in infected granulocyte and macrophage progenitors (GM-Ps—granulocyte–macrophage progenitors), and it starts in a region of 38 bp prior to the initiation site of the cmvIL-10 transcript [21]. However, during the lytic cycle, LAcmvIL-10 initiates at the same initiation site as cmvIL-10. In addition, LAcmvIL-10 is expressed in early kinetics (β), while cmvIL-10 is expressed in late kinetics (γ) [22]. These data lead to the speculation that the transcription of cmvIL-10 and LAcmvIL-10 can occur by activation of different promoters and possibly at different times. Therefore, it is reasonable to hypothesize that the sporadic expression of transcript LAcmvIL-10, observed in our analysis in vivo, may be due to the activation of alternative promoters.

In summary, we showed here the expression pattern of cmvIL-10 and LAcmvIL-10 during early and full reactivation of HCMV in immunosuppressed transplant recipients. We believe that future studies investigating the expression of HCMV *UL111A* transcripts in healthy HCMV seropositive patients with no signs of disease and reactivation could clarify the expression levels of these transcripts during HCMV latency.

## 5. Conclusions

This study shows the expression of two HCMV IL-10 transcripts, cmvIL-10 and LAcmvIL-10, in infected cells in culture and cells from kidney transplant recipients during viral reactivation. Our results indicate that cmvIL-10 is the most expressed transcript during early viral reactivation and productive infection in individuals undergoing kidney transplantation; cmvIL-10 could even be detected in the absence of viral DNA. LAcmvIL-10, on the contrary, was expressed sporadically in a few patients, and it was detected in the absence of or at low levels of viral DNA, reinforcing the findings that it is produced and has important functions during viral latency.

Our data contribute to the understanding of HCMV biology and primarily show how cmvIL-10 and LAcmvIL-10 are expressed during different stages of viral replication, in addition to contributing to future work for establishing HCMV IL-10 monitoring during viral reactivation in infected individuals as a potential diagnostic tool. Importantly, further studies verifying the expression of the other HCMV IL-10 transcripts (C to H) identified in infected cells are necessary to determine if they are expressed during natural infection and if they have specific functions during HCMV infection.

## Data Availability

The original contributions presented in this study are included in the article/supplementary material. Further inquiries can be directed at the corresponding authors.

## Supplementary Materials

The following supporting information can be downloaded at https://www.mdpi.com/article/10.3390/v17040501/s1; Figure S1: Schematic drawing of the primers designed for SYBR Green assay; Figure S2: Tm values obtained in SYBR Green assays; Figure S3: Schematic drawing of the primers and probes designed for TaqMan assay; Figure S4: Amplification of cmvIL-10 and LAcmvIL-10 in a standard curve; Figure S5: Viral DNA in plasma and in PBMCs from patients; Table S1: Primers and probes used for DNA and mRNA detection; Table S2: Clinical data of renal transplant recipients.

## Abbreviations

The following abbreviations are used in this manuscript:

cIL-10	cellular interleukin-10
cmvIL-10	Cytomegalovirus IL-10 (isoform A)
Ct	Cycle threshold
LAcmvIL-10	Latency-associated cytomegalovirus IL-10 (isoform B)
GAPDH	Glyceraldehyde-3-phosphate dehydrogenase
HCMV	Human cytomegalovirus (*Cytomegalovirus humanbeta5*)
hpi	Hours post-infection
IE	Immediate-early
MOI	Multiplicity of infection
NTC	No-template control
PBMC	Peripheral blood mononuclear cells
PMA	Phorbolmyristate acetate
