# Supplementary material for: Expression Profile of Human Cytomegalovirus UL111A cmvIL-10 and LAcmvIL-10 Transcripts in Primary Cells and Cells from Renal Transplant Recipients
Source: Viruses. 2025 Mar 31;17(4):501. doi: 10.3390/v17040501 (PMC12031159; doi:10.3390/v17040501)
Supplement: Supplementary file 1 [file viruses-17-00501-s001.zip › Suppl Material (Tables and Figure Legends).pdf]

Suppl. Material – Tables

Table S1. Primers and probes used for DNA and mRNA detection.

Target	Primer Forward (5′-3′)	Primer Reverse	SYBR/ TaqMan (Probe 5′-3′)
**UL44**	TACAACAGCGTGTCGTGCTCCG	GGCGTGAAAAACATGCGTATCAAC	SYBR
**GAPDH**	ACCCACTCCTCCACCTTTGAC	CTGTTGCTGTAGCCAAATTCGT	SYBR
**cmvIL-10**	AAAACCTACGTTGCAACGTGAGGA	CAACCTAACAGAGGGCATTGC	SYBR
**LAcmvIL-10**	AAAACCTACGTTGCAACGTGAGGA	CTGGGAGCCCGACAAAAGTAAAAC	SYBR
**cmvIL-10**	CGAGTAAAACCTACGTTGCAAC	CCGCAACCTAACAGAGGGCATT	TaqMan (TGTTCCCCGCAGGCGACCAC)
**LAcmvIL-10**	AAAACCTACGTTGCAACGTGAGGA	CTGGGAGCCCGACAAAAGTAAAAC	TaqMan (TGTTCCCCGCAGGCGACCAC)

Table S2. Clinical data of renal transplant recipients.

Transplant recipients	Age (yr)	Gender	Organ rejection[1]	Clinical Data	HCMV DNAemia	Ganciclovir treatment	Viral DNA peak (IU/ml)
**HCMV seropositive**							
**1**	47	M	No	Asymptomatic		No	645
**2**	48	M	No	Asymptomatic	2 episodes	Yes	83340
**3**	51	F	No	Asymptomatic		No	586
**4**	45	M	No	Asymptomatic	2 episodes	Yes	20412
**5**	50	M	No	Asymptomatic	1 episode	Yes	5062
**6**	37	M	No	Asymptomatic		No	2405
**7**	46	F	No	Asymptomatic		No	2087
**8**	50	F	No	Asymptomatic	1 episode	Yes	37978
**9**	33	M	No	Asymptomatic	2 episodes	Yes	7504
**10**	40	M	No	Symptomatic	1 episode	Yes	78
**11**	28	F	No	Asymptomatic	1 episode	No	40
**12**	28	F	No	Asymptomatic	1 episode	Yes	21707
**13**	63	M	No	Asymptomatic	1 episode	Yes	1467
**14**	62	M	No	Asymptomatic	2 episodes	Yes	6187
**15**	47	M	Yes	Asymptomatic	1 episode	Yes	9512
**HCMV Seronegative**							
**16**	37	F	No	Asymptomatic	2 episodes	Yes	1772
**17**	47	M	No	Asymptomatic		No	35

[1] Status at the last collection time-point (60 days post-transplantation)

Figure S1. Schematic drawing of the primers designed for SYBR Green assay. The positions of exons (boxes) and introns (lines) are shown according to HCMV strain TB40e (GeneBank access number KF297339). Primers were designed for exon-exon junctions of cmvIL-10 and LAcmvIL-10 and sequence corresponding to exon in LAcmvIL-10.

Figure S2. Tm values obtained in SYBR Green assays. Graphs A and B show the Tm values obtained after amplification of cmvIL-10 (pink), LAcmvIL-10 B (green) and unspliced (Uns) (red), with primers for cmvIL-10 and LAcmvIL-10. The derived reporter signal (-R) is shown on the Y axis and the Melting temperature (°C) on the X axis.

Figure S3. Schematic drawing of the primers and probes designed for TaqMan assay. The positions of exons (boxes) and introns (lines) are shown according to HCMV strain TB40e (GeneBank: KF297339). Primers for exon-exon junctions are represented by arrows and probes by white boxes.

Figure S4. Amplification of cmvIL-10 and LAcmvIL-10 in a standard curve. Standard curves were generated by amplifying a dilutions series of plasmids containing the cmvIL-10 and LAcmvIL-10 ORFs. Panels A and B show the Cts obtained after amplification of cmvIL-10 and LAcmvIL-10, respectively.

Figure S5. Viral DNA in PBMCs and plasma from patients. The left Y axis shows the relative intracellular viral DNA levels in PBMCs (blue line), using primers for the UL44 gene, normalized by GAPDH gene expression. The $2\text{-}\Delta E*10^6$ is the expression that quantifies the difference between the two genes. The right Y axis shows the viral DNA levels in the plasma in IU/ml (orange line). The X axis shows the days of collection analysis.
