# Supplementary figures and images for: Expression Profile of Human Cytomegalovirus UL111A cmvIL-10 and LAcmvIL-10 Transcripts in Primary Cells and Cells from Renal Transplant Recipients
Source: Viruses. 2025 Mar 31;17(4):501. doi: 10.3390/v17040501 (PMC12031159; doi:10.3390/v17040501)

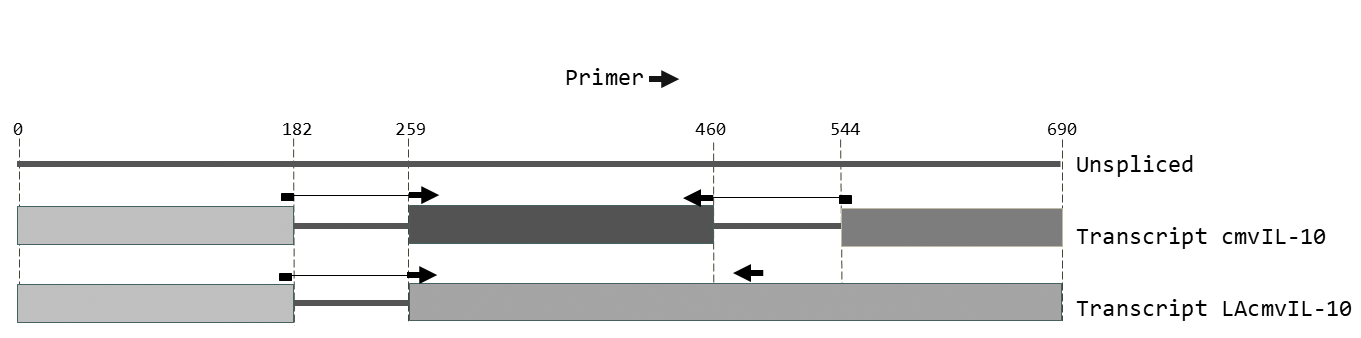

Supplement: Supplementary file 1 [file viruses-17-00501-s001.zip › Figure S1.tif]

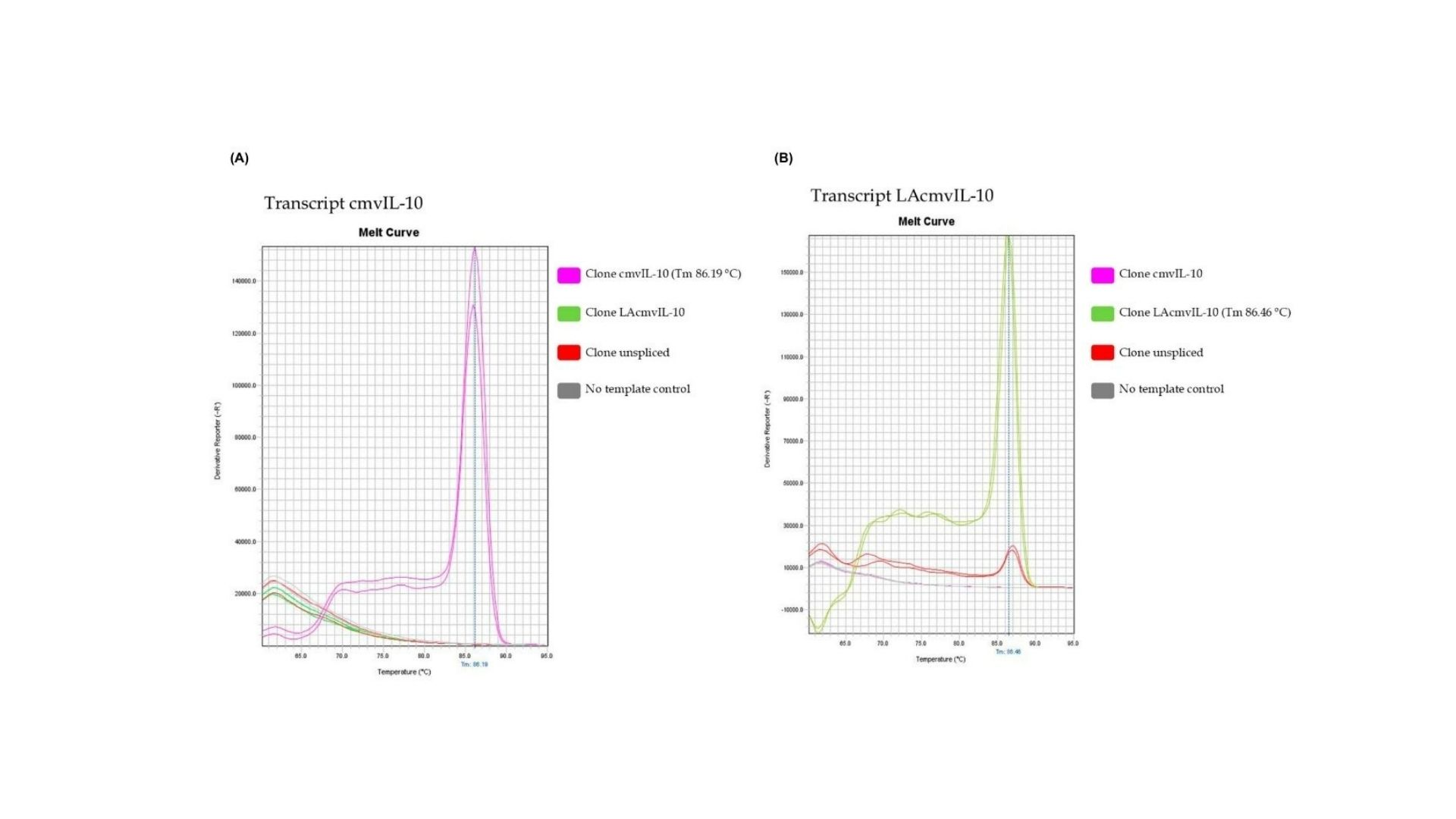

Supplement: Supplementary file 1 [file viruses-17-00501-s001.zip › Figure S2.tiff]

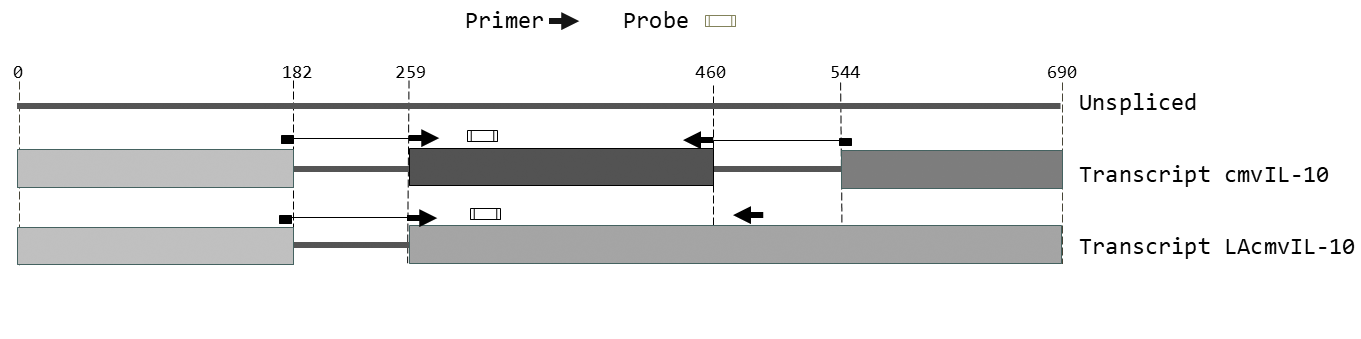

Supplement: Supplementary file 1 [file viruses-17-00501-s001.zip › Figure S3.tif]

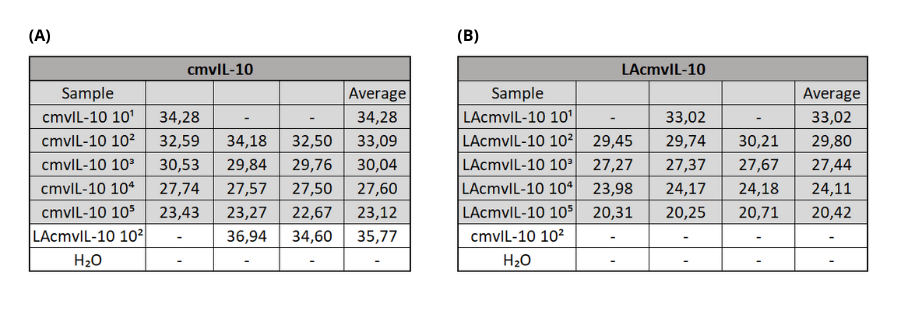

Supplement: Supplementary file 1 [file viruses-17-00501-s001.zip › Figure S4.tif]

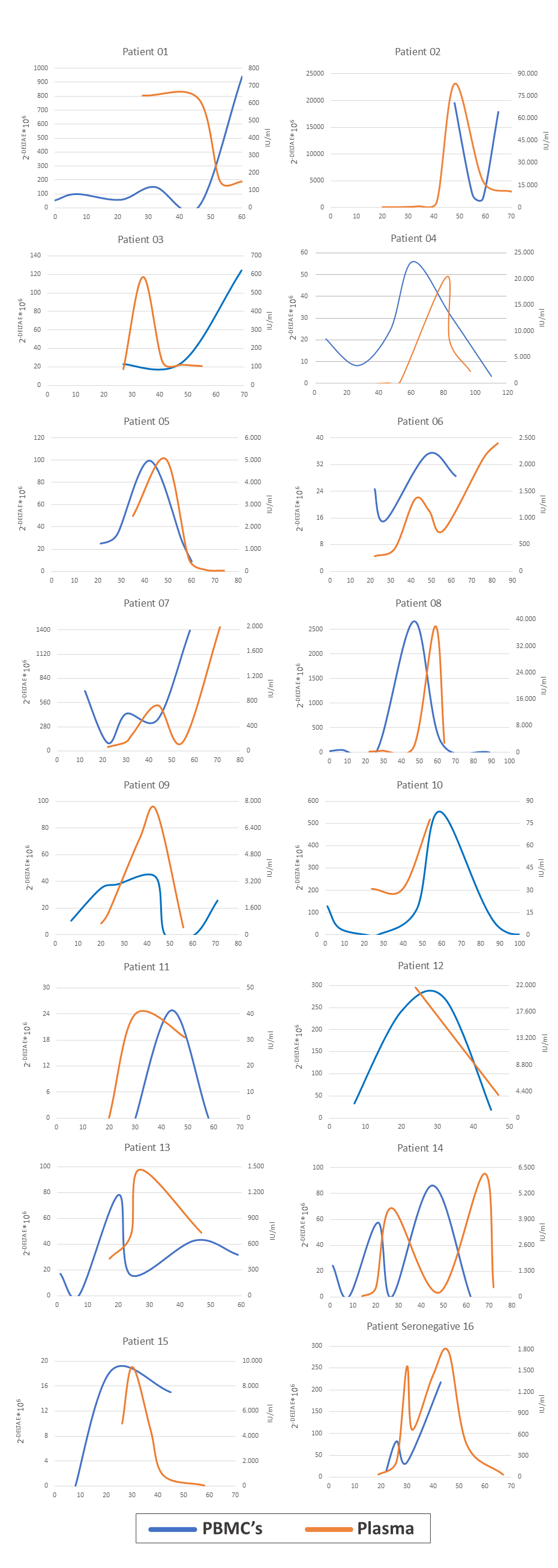

Supplement: Supplementary file 1 [file viruses-17-00501-s001.zip › Figure S5.tif]
